# Under-Etched Plasmonic Disks on Indium Tin Oxide for Enhanced Refractive Index Sensing on a Combined Electrochemical and Optical Platform

**DOI:** 10.3390/ma13040853

**Published:** 2020-02-13

**Authors:** Hans Dyrnesli, Gunnar Klös, Duncan S. Sutherland

**Affiliations:** Interdisciplinary nanoscience center (iNANO), Aarhus University, 8000 Aarhus, Denmark; hansdyrnesli@gmail.com (H.D.); gunnar.klos@chem.ox.ac.uk (G.K.)

**Keywords:** plasmonics, refractive index sensor, colloidal lithography

## Abstract

A simple approach to enhance the refractive index sensitivity of gold nanodisks immobilized on electrically conducting indium tin oxide (ITO) substrates has been demonstrated. A two-fold increase in sensitivity to bulk refractive index change was achieved by substrate under-etching of gold nanodisks on ITO in 50 mM sulfuric acid. The influence of an intermediate titanium adhesion layer was investigated and was found to markedly influence the etching pattern and time. Etching with an adhesion layer resulted in enhanced refractive index sensitivity on disk-on-pin like structures after long etching times, whereas etching of disks deposited directly on ITO resulted in a disk-on-pincushion like configuration and similarly enhanced sensitivity already at shorter times. The gold disks remained electrically connected to the ITO substrate throughout etching and allowed site-specific electrodeposition of poly(3-aminophenol) at the nanodisks, showing enhanced thin-film refractive index sensitivity. This work demonstrates a simple method for enhancing refractive index sensitivity of nanostructures on ITO substrates for combined electrochemical and optical platforms, and subsequently a method to modify the surface of the electrically connected nanostructures, which has potential application in biosensing.

## 1. Introduction

Plasmonic nanostructures have been the subject of intense research interest due to their potential application in biological/chemical sensing [1], and as nanoantennas and nanolenses for manipulating light below the diffraction limit [2]. Interactions of light with sub-wavelength nanoparticles of specific metals (e.g. Ag, Au, and Al), are dominated, in particular spectral regions in the UV, visible and NIR, by incident field-driven oscillations of conduction electrons spatially confined to the nanoparticles. The resulting standing oscillations are known as localized surface plasmon resonances (LSPRs), and the displaced electrons cause large field enhancements near the nanoparticle surfaces. The spectral position of LSPR modes are dependent on particle material, shape, size, inter-particle coupling, and dielectric properties of the surrounding medium [1,3,4,5]. Noble metal nanoparticles generally support LSPRs at optical frequencies, and specifically gold has been widely used because of its attractive optical properties, high chemical stability, and ease of functionalization. The sensitivity of the plasmon resonance spectral position to surrounding dielectric properties allows the use of LSPRs for refractive index sensing with a wavelength-based sensitivity that is increased for nanostructures with longer wavelength resonances [5,6]. For many applications, it is advantageous to immobilize plasmonic nanoparticles on a substrate. Immobilization allows control over particle spacing and orientation, which is of fundamental importance in, for example, nanolensing [2], LSPR-based directional scattering [7] and manipulation of electrical potential for use in electrochemistry [8]. The electrically conductive nature of noble metal nanoparticles offers the possibility of combining electrochemical and optical sensing capabilities on a single substrate, and in combination with transparent conducting oxides (TCOs) allow for convenient optical studies of electrochemically induced perturbations in resonance conditions [9,10]. Electrochemical processes at metallic surfaces, including micro and nanostructures, are advantageous in the preparation of sensing devices with polymeric ligands (e.g., molecular imprint polymers—MIP) [11,12]. MIP layers can be prepared at plasmonic surfaces by bulk polymer synthesis [13] but control of film growth is difficult. Electropolymerization is in principle a better approach for plasmonic sensors as it can match the sensor layer thickness to the near-field of the plasmonic nanostructure. Immobilization of nanoparticles/nanostructures at conducting surfaces can enable direct electrochemical access, however for plasmonic refractive index sensing a drawback of immobilized nanoparticles compared to nanoparticles in solution is the substrate-induced reduction in refractive index (RI) sensitivity [5,6,14]. The substrate can affect the sensing performance of nanoparticles by limiting the exposed surface area of the metal, and by damping the LSPR. Damping results in resonance broadening, and is dependent on the substrate material and the degree of spatial overlap between the substrate and the enhanced near field [15,16]. By manipulating these parameters, substrate effects can be minimized. Indeed, some of the highest sensitivities reported in literature have been achieved by minimizing the contact area between nanoparticles and substrates [6,17]. 

Here, we have demonstrated a simple method for substantially increasing the RI sensitivity of gold nanodisks immobilized on an indium-tin oxide (ITO) substrate. We decreased the contact area between gold nanodisk and substrate through wet etching of substrate while retaining electrical connectivity. The use of in-situ characterization of the plasmonic resonances during the etch process and comparison to ex-situ structural characterization by scanning electron microscopy gave insight into the etching process. Gold disks attached to the surface via titanium adhesion layers formed a disk on pin configuration with increasing sensitivity of the plasmon resonance to refractive index changes. For gold disks without a titanium adhesion layer, similar RI sensitivities to those measured for the pin on disk configurations were achieved, both at significantly reduced etching times and while maintaining electrical contact to the plasmonic structures. We demonstrated the electrical connectivity to the nanodisks by electropolymerization of a polymer layer at the plasmonic nanostructure. Near-field interactions were investigated by measuring the optical response to sequential electropolymerization steps of 3-aminophenol on the gold nanodisks, which confirmed a higher spectral shift for disk on pin configurations. The approach has the potential to be applied to the fabrication of combined plasmonic/electrochemical sensors.

## 2. Materials and Methods

All solvents and chemicals were purchased from Sigma Aldrich (Denmark), unless otherwise stated, and were of analytical grade. Solutions were prepared using ultra pure deionized water (DI, resistivity >18 MΩ∙cm).

### 2.1. Gold Nanodisk Fabrication 

**Figure 1:**
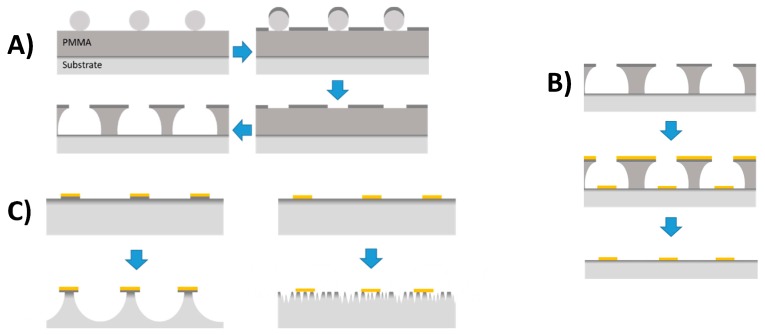
Schematic representation of nanodisk fabrication using hole-mask colloidal lithography. (**A**) Polystyrene particles self-assembles onto PMMA coated ITO substrates, followed by titanium mask deposition, tape stripping, and plasma etching. (**B**) Gold is evaporated through the mask at normal angles either directly onto the substrate (illustrated here), or following deposition of a 2-nm titanium adhesion layer. Subsequent mask removal leaves arrays of gold nanodisks on substrate. (**C**) Under-etching of substrate for gold nanodisks immobilized on ITO with (left) or without (right) an intermediate titanium adhesion layer.

Gold disks with a nominal diameter of 100 nm were deposited on ITO-coated borosilicate glass slides (Deposition Research Lab, St Charles, USA) using hole-mask colloidal lithography (HCL) [18]. Briefly, 160–180 nm of poly(methyl methacrylate) (A495 PMMA, Kayaku Advanced Materials, USA) was spun-coated onto ITO-glass followed by sequential adsorption, rinsing and drying of three layers of polyelectrolytes (poly(diallyldimethylammonium chloride) (PDDA) 2%, poly(styrenesulfonate) (PSS) 2% and polyaluminium chloride (PAX-XL60, Kemira Miljø A/S,Esbjerg, Denmark) 5%, all in DI). Sulfonated polystyrene particles (0.2% by weight in DI) of 0.1 µm nominal size (Thermo Fischer Scientific, Denmark) were self-assembled onto the surface for 2 min, and excess particles were removed by thorough rinsing with DI. Once dried under a stream of nitrogen, a 20-nm sacrificial titanium mask was deposited by physical vapor deposition (PVD) electron beam induced thermal evaporation (Cryofox GLAD, Polyteknik A/S, Østervrå, Denmark). Titanium-capped polystyrene particles were removed by tape-stripping, and oxygen plasma (RF power 50 W, 25 mbar, 40 sccm oxygen) was used to etch exposed PMMA down to the ITO-coated glass substrate (Figure 1A). Through the mask, 20 nm gold was evaporated via PVD at normal angle and the mask removed by sequential sonication in acetone, ethanol, and DI, leaving gold disks with a nominal diameter of 100 nm and a height of 20 nm on the substrate surface (Figure 1B).

### 2.2. Substrate Etching

Substrate etching was performed in a 50 mM solution of H_2_SO_4_ under very gentle flow (Figure 1C). For the etch assay, gold nanodisk-patterned ITO substrates were mounted in a home made optical/electrochemical flow cell with an exposed surface area of 0.64 cm^2^ and a cell volume of 190 µL. Flow was established by a syringe pump, and was set to 250 µL/hour. Samples for electropolymerization were etched by submersion in a 25 mL beaker containing 50 mM H_2_SO_4_.

### 2.3. Electrochemistry

All electrochemical measurements were made using an Ivium CompactStat in a three-electrode configuration connected to a laptop running the latest version of IviumSoft. Gold nanodisk-patterned ITO substrates were cleaned by sequential ultrasonication (or rinsing for etched samples) in acetone, ethanol, and MQ for 10 min and dried under a stream of N_2_. The clean samples were then used a working electrodes in a home-made optical/electrochemical flow cell with a sample volume of 190 µL, a platinum wire counter electrode (99.99%, 0.5 mm, Sigma), and a sealed PTFE Ag/AgCl (3M KCl) reference electrode (eDAQ, ET072). Electro polymerization was performed by cyclic voltammetry in 500 µM 3-aminophenol in 10 times diluted phosphate buffered saline (PBS), scanning between −250 mV and 850 mV at 50 mV/s. Electrochemical impedance spectroscopy measurements were made at open circuit potential in aqueous 5 mM hexacyanoferrate (II/III) with a superimposed sinusoidally varying potential of 10 mV amplitude at frequencies between 0.1 Hz and 10 kHz.

### 2.4. Characterization

Nanostructured surfaces were imaged using scanning electron microscopy (FEI Magellan 400, 5 kV, and 50 pA emission current), and all stated dimensions established using ImageJ. Optical spectra were recorded using a scanning UV–VIS–NIR spectrophotometer (Shimadzu UV-3600, only for 44 hours etch assay) or a fiber-coupled photodiode array spectrophotometer (BWTek Cypher H) with a tungsten light source (BWTek BPS2.0). Extinction was calculated as log (reference intensity/sample transmitted intensity) at each wavelength and plotted vs. wavelength. Borosilicate glass slides were used as references.

### 2.5. Simulation

Simulations were performed with the Finite-Difference Time-Domain (FDTD) solver from Lumerical Solutions (Canada). The dielectric function of Au were taken from Johnson & Christy [19], that for ITO from [20]. A minimum mesh size of 2 × 2 × 2 nm^3^ was used with perfectly matched layer boundary conditions.

## 3. Results and Discussion

**Figure 2:**
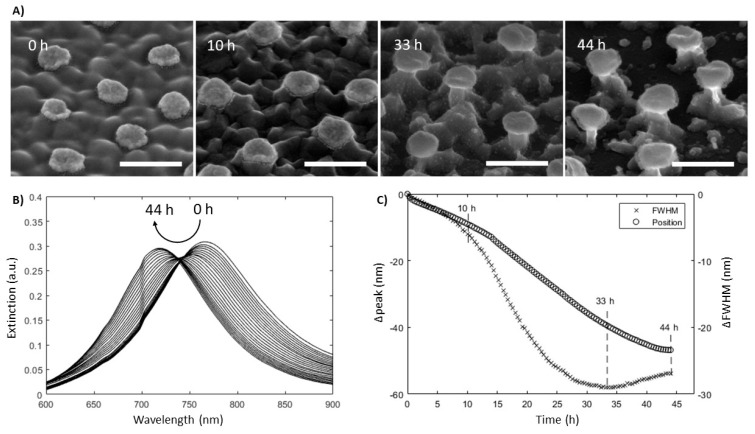
Etch assay of gold nanodisk-patterned ITO substrates. (**A**) SEM images at selected times during etching taken at a 45° angle showing evolution of gold disks on ITO pin-like structures. 44 h-sample was coated with 5 nm titanium, because the ITO film was etched down to the underlying glass substrate, breaking electrical conductivity in the film. Scale bars are 200 nm. (**B**) Raw spectra showing gold nanodisk resonance peaks during etching. Individual spectra are spaced two hours apart. The curved arrow shows evolution of the peak positions. (**C**) Spectral shift and width of the plasmonic resonance plotted as a function of the etching time. The width reaches a minimum around 33 h into the etch assay. The peak position appears to reach a minimum after 44 h.

**Figure 3:**
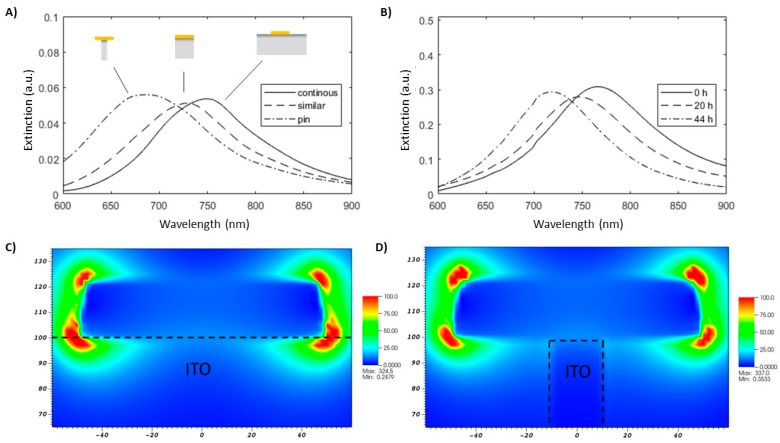
Extinction Spectra and plots of local electric field at resonance for metallic nanodisks supported at a layer or at a pillar of ITO. (**A**) Calculated spectra for 100 nm diameter 20 nm tall truncated cone gold nanodisks (2 nm Ti Adhesion layer) in water (n = 1.33) supported by a continuous ITO layer or by a 100 nm tall × 20 nm wide ITO pillar respectively. (**B**) Corresponding recorded experimental spectra for gold nanodisks on ITO after 0, 20, and 44 h of etching, respectively. (**C**) Calculated field enhancement at resonance for gold nanodisks on continuous ITO (C) and 20 nm wide pillars of ITO (**D**).

20 nm tall gold nanodisks with a diameter of 104 ± 5 nm and with a 2 nm titanium adhesion layer were fabricated on ITO substrates. Figure 2A (0 h) shows a SEM image of the fabricated disks viewed from a 45° angle. The nanodisk-patterned ITO substrate was mounted in an optical flow cell and etched for a total of 44 h in a gentle flow of 50 mM H_2_SO_4_ while continuously recording the optical spectrum. The resulting optical data are shown in Figure 2B,C along with SEM images taken at various times during etching (A). A total blue-shift of the gold nanodisk resonance peak of approximately 47 nm is observed after 44 h, accompanied by a substantial decrease in peak width (19% decrease at minimum around 33 h) as seen in Figure 2C. The general evolution of the wavelength of the plasmonic resonance was replicated in simulations of gold nanodisks on ITO substrates of varying dimensions, but not the decrease in width of the resonance peak (Figure 3A simulations, Figure 3B experiments). This discrepancy between the exeperiment and the simulations suggests that the observed change in experimental spectral peak width was related to effects other than reduced damping by the substrate. One contribution to this behavior could be the result of chemically mediated recrystallization, which has been observed in gold films, albeit under much harsher oxidative conditions [21]. In that work, recrystallization involved a size increase of grains in polycrystalline structures. Dahlin et al. reported that sharpening and a blue-shift of the resonance peak for gold nanodisks during electrochemical recrystallization and interpreted is originating from a reduced electron scattering at grain boundaries of the larger grains, overall reduced electron effective mass, and an increase in apparent electron density [9]. Another likely explanation for the difference between experimental spectral widths and simulations is the inhomogeneous broadening of the plasmonic resonance due to either variations in size/aspect ratio of the nanodisks or in the Au-ITO interactions as well as the the high surface roughness, which is not accounted for by the simulation model. SEM images (Figure 2A) suggest that some smoothening of the nanostructure can occur after longer etching times and may have led to a reduced variation in the detailed shape of the structures across the array. Additionally morphological variations of the substrate near the sensitive regions of the nanodisks may be reduced, resulting in a more monodisperse substrate interaction. Both effects could contribute to a reduction of the spectral peak width (Figure 2A). Figure 2C shows the spectral shift and width of the plasmonic resonance plotted as a function of the etching time and an increase of downward slope was observed between 7 and 15 h into the etch process which may indicate a shift in the etch pattern (Figure 2C). The sensitive regions around the nanodisks (as represented by the high field sites in Figure 3C) are localized at the corners of the plasmonic structure. The etch rate of ITO across this sensitive region will depend on the exact etch profile and as cavities are etched in the substrate between the disks, the exposed surface area likely increases increasing the lateral etch rate. Temperature fluctuations during the etch assay may have influenced the etch rate, as no measures were taken to maintain the system at a constant temperature.

The spectral peak width reaches a minimum around 33 h into the etch assay, after which a gradual broadening is observed. This is believed to be caused by orientational changes of some of the gold nanodisks as the support pillar gets very thin or fully etched, resulting in increased inhomogeneous broadening of the resonance peak. 

**Figure 4:**
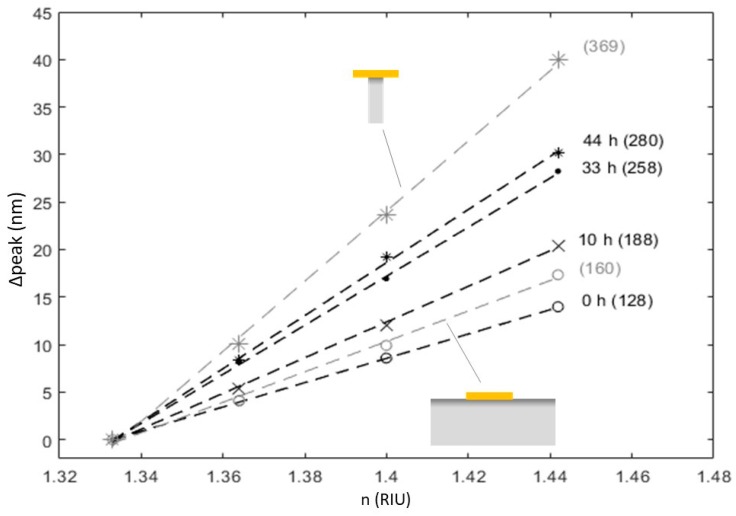
Spectral shift of the plasmonic resonance in response to bulk refractive index change. Shifts of the measured peak positions in response to RI change are presented for gold nanodisk-patterned ITO substrates after 0, 10, 33, and 44 hours of etching along with best linear fits (dashed lines). Shown in grey are calculated peak shifts for disks on continuous and pin-shaped ITO substrate, respectively. Brackets show RI sensitivity (dλ/dn).

The sensitivity of the spectral resonance of the nanodisk-patterned ITO substrates to bulk refractive index changes after 0, 10, 33, and 44 h of etching was measured by injecting aqueous sucrose solutions of increasing concentrations, corresponding to refractive index values ranging between 1.33 and 1.44, into the flow cell. Raw data and best linear fits are presented in Figure 4 along with the calculated response of gold disks on 20 nm wide 100 nm tall ITO pins, and on continuous ITO, respectively. A roughly linear red shift of the resonance peaks was seen as the bulk refractive index was increased, which is in agreement with literature [22]. The measured refractive index sensitivity for disks at flat ITO surfaces (128 nm/RIU) is comparable to that reported for gold nanostructures immobilized at flat non-conducting surfaces at this wavelength e.g., gold disks immobilized on glass 130 nm/RIU for a 690 nm wavelength resonances [5]. Improved refractive index sensitivities have been achieved by reducing the effect of the substrate e.g., raising nanodisks on pillars (290 nm/RIU at 700 nm) [5] or in a disk-on-pin configuration on glass (222 nm/RIU at 675 nm resonance) [23]. The RI sensitivities measured for the structures in this work should be compared to literature values for other systems which allow electrical access to the plasmonic structures. Two types of electrical access for localized plasmonic systems have been reported utilizing either continuous metal films with arrays of localized plasmonic elements (e.g., nanoholes or nanocups) or discrete metal nanoparticles on transparent conducting films (such as ITO). LSPR’s at gold nanohole arrays on glass showed 125 nm/RIU for a 700 nm wavelength resonance [24] while gold nanocup arrays showed ~100 nm/RIU at a resonance wavelength of 620 nm [25]. Compared to isolated nanostructures [5] a more modest enhancement was seen for raising conducting films away from the surfaces (e.g., raised nanohole arrays—160 nm/RIU at 700 nm) [24]. One report describes the RI sensitivity for isolated gold structures in an island film array on an ITO substrate and showed a maximum refractive index sensitivity of 217 nm/RIU at 600 nm resonance wavelength [26]. Metal island films provide simple means to produce plasmonically active surfaces but typically give significant inhomogeneous broadening of the resonance peaks through size/shape variation of the structures and coupling between adjacent structures. To allow direct comparison of the performance of nanostructures in refractive index sensing, Sherry et al. introduced the figure of merit (FOM) [27]. The FOM is a size- and shape-independent dimensionless number given the ratio between the slope of the refractive index change curve and the resonance line width. From the sensitivities reported in Figure 4 and the corresponding resonance peak widths in water, the FOMs obtained were 0.9 and 1.2, for measured and simulated response of gold nanodisks on continuous ITO, respectively. FOM values were 2.2 and 2.5 for measured and simulated response of gold nanodisks on the pin-like ITO structures, showing a clear increase in performance and values significantly higher than that of the previously reported gold island films on ITO [26], which had a maximum FOM of ~1.4–1.5. The lower FOM of the gold island films comes from the broader resonance of ~130nm (or 0.58 eV at the resonance) for the island films compared to ~110nm (or 0.26 eV at the resonance) for the structures studied here, likely from inhomogeneous broadening and coupling between adjacent nanostructures.

We demonstrated here an approach to reduce the effect of the substrate in the disk on pin format [23] while retaining electrical access. The refractive index sensitivity for the disk on pin structures for the long etched samples studied here reached 269 nm/RIU for a resonance peak at 720 nm with FOM of 2.2, which represents the highest reported sensitivity for electrically addressable localized surface plasmon resonances. The plasmon resonances are localized to individual disks and could be applied to small area or single disk measurements. 

Simulated data yielded higher refractive index sensitivities than the corresponding experimentally obtained values, which in the case of the disk on pin were comparable to the maximum sensitivity obtained for gold plasmonic structures at this spectral position [5], indicating that the disks were decoupled from the substrates. The refractive index sensitivity for a plasmonic structure on a substrate is expected to be sensitive to the fraction of the near field that is buried in the substrate [6]. In electrodynamic simulations, the specific calculated distribution of the near field is strongly influenced by the detailed shape of the nanostructure and substrate and the shapes vary somewhat across the distribution, which likely explains the origin of the systematic difference between the calculated refractive indexes and experimentally determined values. However, the overall effect of increased refractive index sensitivity with etching resulting from localized removal of substrate is clear. An additional systematic artefact could be a difficulty to represent the very thin titanium adhesion layer with finite size discrete block. The sensitivity discrepancy between the simulated gold disk on an ITO pin and the value obtained experimentally for gold nanodisk-patterned substrates after 44 h of etching is additionally likely due to an underestimation of the substrate influence by the simulation model. Firstly, a pin width of 20 nm, estimated by the thinnest sections of the pins from SEM images, might be a low estimate for a representative average (Figure 2C, 44 h). Secondly, rather than a cylinder with uniform diameter, the pin might be better approximated by an elongated hourglass-like pedestal, which, having significantly larger contact area with the gold nanodisks, would reduce the sensitivity to RI change.

While the use of titanium adhesion layers for plasmonic nanostructures is advantageous in terms of robustness of the system it creates a multilayer of different materials altering the local dielectric distribution around the plasmonic nanostructure and complicating numerical simulations of the optical properties. PVD-deposited gold structures adhere well to ITO, which has recently been proposed as a superior, less resonance-broadening alternative to titanium adhesion layers for gold nanoparticle-based optical devices [28,29]. Here the substrate around the plasmonic sensing element has been removed to enhance the refractive index sensitivity; however, a remaining adhesion layer may act as substrate, reducing the benefit.

To avoid the adhesion layer, gold nanodisks were fabricated directly on ITO substrate. Gold nanodisk-patterned ITO substrates without titanium adhesion layer were subsequently etched in a 50 ml beaker of stagnant 50 mM H_2_SO_4_ rather than in a flow cell. Initially, bulk etching was performed on a sample until disks were observed to detach, and the etching time was then reduced for subsequent samples. Etching for 5.5 h under ambient conditions gave consistent results, and the resulting under-etched gold nanodisks had a sensitivity to bulk refractive index change was 260 nm/RIU similar to that found for disks on pins (~280 nm/RIU), and roughly twice that of the non-etched sample (~130 nm/RIU). In Figure S1, the large increase in sensitivity after etching indicates that the substrate around the high field sites must have been significantly removed.

We demonstrated the electrical connectivity of the gold nanostructures and explored their near-field refractive index sensitivity to a thin-film dielectric layer using sequential depositions of poly(3-aminophenol). 3-aminophenol is an aromatic amine and alcohol capable of polymerizing through oxidation of its amine [30,31], which has previously been applied to the elecropolymerization of molecular imprint polymers [31]. Electropolymerized poly(3-aminophenol) films are non-conducting, and growth is thus self-limiting [30]. 

**Figure 5:**
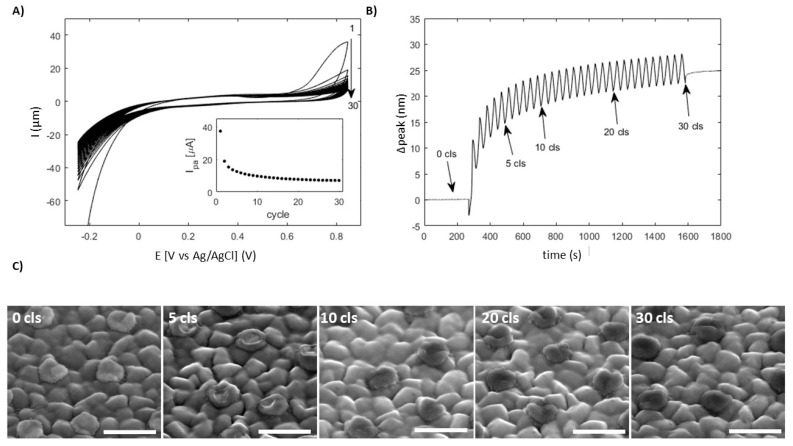
Poly(3-aminophenol) deposition on gold nanodisk-patterned ITO substrate without titanium adhesion layers. (**A**) Cyclic voltammograms for 30 continuous cycles of electropolymerization. Measured current at 850 mV as a function of deposition cycle is shown in the insert. (**B**) Resonance peak position as a function of time during deposition. Periodic variation is due to effect of the applied potential on the electron density of the gold nanodisks. (**C**) SEM images of gold nanodisks after selected numbers of deposition cycles. Diameter increase after 30 cycles is roughly 23 nm. Scale bars are 200 nm.

3-aminophenol was polymerized by cyclically scanning the potential of the unetched gold disk-patterned ITO substrate between -250 mV and 850 mV in 500 µM 3-aminophenol in 1:10 diluted PBS (Figure 5A). The peak current of the oxidation wave around 850 mV shows a rapid decrease after the first few cycles, followed by a slow and more gradual decrease (insert of Figure 5A). This is consistent with a potential drop caused by passivation of the gold nanodisk-patterned electrode by the resulting poly(3-aminophenol) film, resulting in less deposition in subsequent cycles, which has previously been reported for deposition of poly(3-aminophenol) on gold electrodes [32].

The plasmon resonance of the gold nanodisk shifts to longer wavelength over time during the polymer electrodeposition cycles (Figure 5B). The rate of the red shift matches the rate of anodic peak current reduction (Figure 5A insert) and has a superimposed periodic periodic variation (Figure 5B). The overall shape will result from a sum of two competing processes. Firstly, deposition of dielectric poly(3-amiphoenol) (n=∼1.7 at 546.1 nm for similar poly(2-aminophenol) films [33,34] film) on the gold structures will cause a red-shift of the resonance peak. Secondly, electrochemical recrystallization of the gold nanodisks can cause a blue shift of the resonance peak due to crystal grain growth, an effect previously reported for gold nanodisks subjected to similar potentials [9]. The effect is clearly visible in Figure S2 showing the gradual shift toward shorter wavelengths caused by potential scan in buffer. However, the effect is small compared to the sensitivity of the gold nanodisks to refractive index change. The observed superimposed periodic variation is caused by a potential-induced perturbation of the electron density of the gold nanodisks [8], resulting in varying resonance with a periodicity defined by the parameters of the triangular potential versus time waveform used in the cyclic voltammetry deposition. 

Poly(3-aminophenol) deposition appears localized to the gold nanodisks, as is seen in the SEM images of the patterned ITO substrate after 30 cycles of deposition (Figure 5C). This localization is further supported by the only minor increase in charge transfer resistance across the deposited patterned ITO interface observed in electrochemical impedance spectra (Figure S3), and is likely due to slow reaction kinetics on the bare ITO surface. Deposition starts at the edges of the gold nanodisks, where the field resulting from the applied potential is likely to be concentrated, as seen by the ring-like coverage after five cycles (Figure 5C). This area coincides with the region of highest field-enhancement for the gold nanodisks at the lowest order resonance (Figure 3C), and causes the relatively large shift of the resonance peak observed in the first few deposition cycles in Figure 5B.

**Figure 6:**
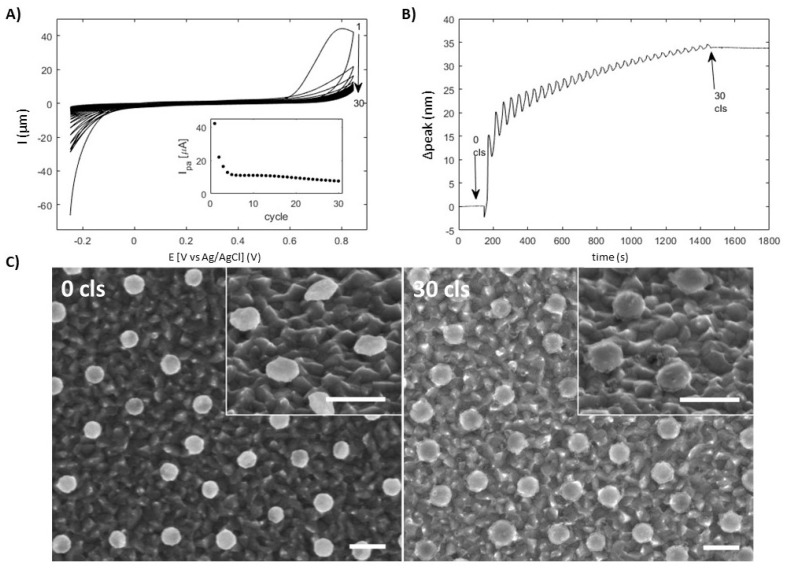
Poly(3-aminophenol) deposition on under-etched gold nanodisk-patterned ITO substrate without titanium adhesion layer. (**A**) CV curves for 30 cycles of electropolymerization with current at 850 mV as a function of deposition cycle (insert). (**B**) Resonance peak position as a function of time during deposition. (**C**) SEM images of under-etched gold nanodisks before and after 30 deposition cycles. Inserts are taken at an angle of 45°. Diameter increase after 30 cycles is roughly 21 nm. Scale bars are 200 nm.

An example of the etched gold nanodisk-patterned ITO surface (with no titanium adhesion layer) can be seen in Figure 6C (0 cls). The etching process in the absence of a titanium adhesion layer led to large increases in refractive index sensitivity after only 5.5 h (compared to 33 h etching to reach similar sensitivity for gold nanodisks with a titanium adhesion layer. The overall shorter etch time does not give sufficient time to remove the bulk of the material of the ITO between the disks so that the these structures (Figure 6C (0 cls)) do not show the disk on pin configuration of the longer etched structures (Figure 2C, however the SEM image (inset Figure 6C) shows under-etching of the gold film. The degree of roughening of the ITO surface qualitatively suggests that the etch rate is similar to that observed for the exposed substrate in Figure 2 for nanodisks deposited with a titanium adhesion layer, and that the flow rate was not limiting.

The titanium adhesion layer appears to alter the etching of the ITO close to the gold surface. Titanium is a highly reactive metal that forms strong chemical bonds with oxygen species on the substrate surfaces, and enhances wetting of the subsequently deposited gold [35] and we suggest that it prevents etching along the facets of ITO in contact with the nanostructure. We propose that the enhanced bulk refractive index sensitivity of gold nanodisks at shorter etch times without a titanium adhesion layer compared to with an adhesion layer results from interfacial etching of ITO/Au contact surface in the absence of an adhesion layer. 

Poly(3-aminophenol) thin films were sequentially deposited on the under-etched gold nanodisks to compare the near-field sensitivity for thin films measured at under-etched disks. Figure shows the resulting spectroelectrochemical data along with SEM images of the disks before and after poly(3-aminophenol) deposition. The overall profile is similar to that for 3-aminophenol deposition at unetched samples (Figure 5A,B). The plasmon resonance of the gold nanodisk shifts to longer wavelength over time during the polymer electrodeposition cycles with an oscillation resulting from the sweeped potential during the electrodeposition cycles. Notably, the wavelength shift of the plasmon resonance during the first cycle is roughly one-third larger for the etched sample than that of the non-etched sample, which fits qualitatively with the large bulk refractive index sensitivity observed (Figure S1 in Supplementary Materials). The amplitude of the oscillation of the spectral resonance position, which is superimposed on the general red shift is seen to decrease over time for the etched samples but not for the unetched samples. This oscillation is thought to result from potential induced modulation of the electron density in the metal. The reducing amplitude with increasing electrodeposition cycles suggests that fewer and fewer disks are electrically connected or less well connected over time. We interpret this as the electrodeposition below the disks reducing or preventing electrical connection leading to smaller or no changes in electron density of a majority of disks which may interfere with processes electrodepositing thick layers. This approach of make site specific deposition of electropolymers on plasmonic nanostructures represents a novel approach to investigate the extent of the near-field from the metal surface either by a cyclic layer by layer deposition or by continuous deposition. In principle, the deposition could be limited to specific regions of a nanofabricated plasmonic structure by the use of dielectric protective coatings (e.g., silica disks placed on top of gold disks to limit access to the disk edge). Electrical access to plasmonic structures has been used in several applied and scientific studies [9] in sensing and biosensing [25,36] and catalysis [37,38]. The approach presented here provides a means to reduce the effect of the surface (to decouple the plasmonic structure from the surface [17]) while retaining electrical contact via ITO films, which may be applied in such studies and applications.

## 4. Conclusions

In this work, we have studied approaches to increase the refractive index sensitivity of plasmonic nanostructures immobilized on substrates by removing parts of the substrate. We demonstrated a simple method for increasing the sensitivity of gold nanodisks immobilized on ITO substrate. The use of titanium adhesion layers lead to disk on pin configurations after etching with increased refractive index sensitivity. Interestingly, equally large increases of bulk refractive index sensitivity could be achieved by shorter etching times by rapid and non-isotropic under-etching of the polycrystalline substrate while keeping contact points for electrical connection. The plasmonic disks were electrically connected through the ITO substrates, which allowed for manipulation of the potential on the gold nanodisks. This property enabled sequential electrochemical growth of poly(3-aminophenol) specifically on the gold nanodisks as a novel way of investigating near-field interactions. Similar aminophenols are used in formation of molecularly imprinted polymers (MIPs), also known as plastic antibodies, in which highly stable cavities of template molecules are imprinted in a polymer matrix, imparting affinity towards the template molecule on the MIP [39,40,41]. The work presented here could provide an attractive and sensitive platform for selective functionalization and nanofabrication for use in optical and electrochemical sensing.

## Supplementary Materials

The following are available online at https://www.mdpi.com/1996-1944/13/4/853/s1. Figure S1—Real-time changes in resonance peak position during changes in bulk refractive index; Figure S2—Real-time changes in resonance peak position during potential cycling; Figure S3—Electrochemical impedance spectra before and after electrodeposition of layers of 3-aminophenol.
