# Supplementary material for: Under-Etched Plasmonic Disks on Indium Tin Oxide for Enhanced Refractive Index Sensing on a Combined Electrochemical and Optical Platform
Source: Materials (Basel). 2020 Feb 13;13(4):853. doi: 10.3390/ma13040853 (PMC7078751; doi:10.3390/ma13040853)
Supplement: Supplementary file 1 [file materials-13-00853-s001.pdf]

materials

MDPI

Article

Under-Etched Plasmonic Disks on Indium Tin Oxide for Enhanced Refractive Index Sensing on a Combined Electrochemical and Optical Platform

H. Dyrnesli, G. Klös, and D. S. Sutherland *

Interdisciplinary nanoscience center (iNANO), Aarhus University, 8000 Denmark; hansdyrnesli@gmail.com (H.D.); gunnar.klos@chem.ox.ac.uk (G.K.)

* Correspondence: duncan@inano.au.dk

Received: 17 December 2019; Accepted: 12 February 2020; Published: date

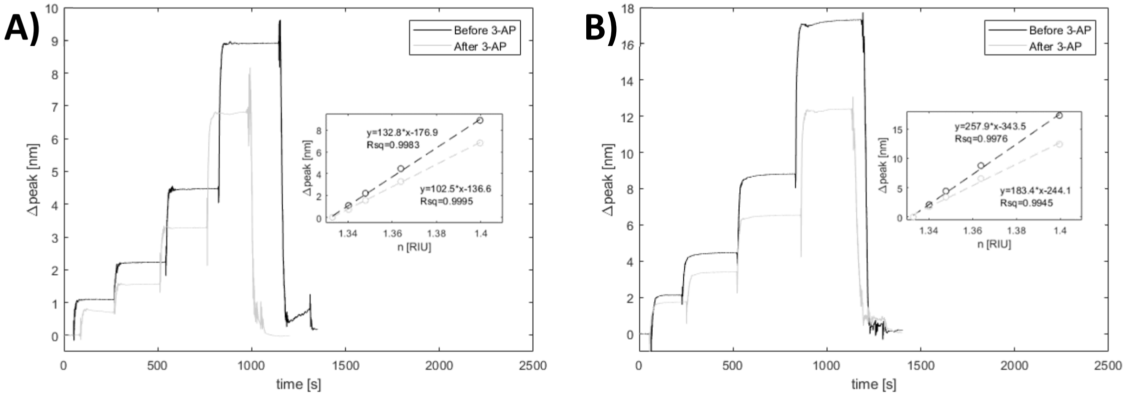

Figure S1. Change in resonance peak position in response to bulk refractive index change for non-etched (**A**) and under-etched (**B**) gold nanodisks before (black) and after (grey) 30 cycles of poly(3-aminophenol) deposition, respectively. Inserts show best linear fit.

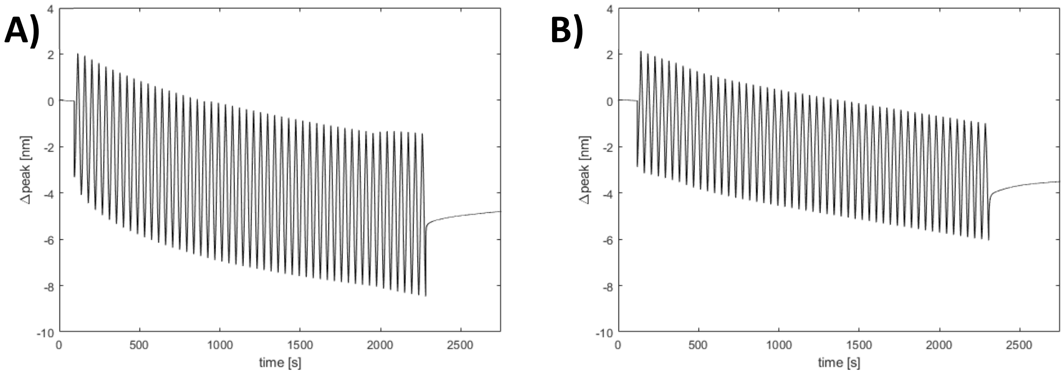

Figure S2. Resonance peak shift during potential cycling in ten times diluted PBS for unetched (**A**) and etched (**B**) samples of gold nanodisks deposited directly on ITO.

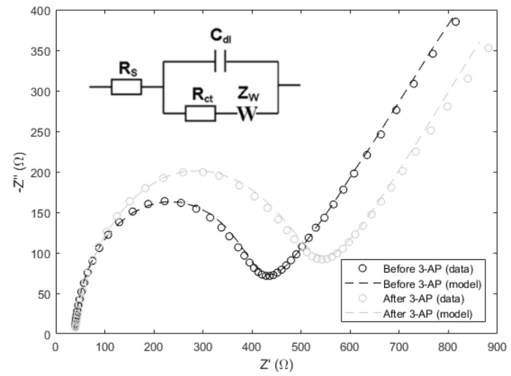

Figure S3. Electrochemical impedance spectra before (black) and after (grey) 30 deposition cycles of poly(3-aminophenol) on an unetched sample of gold nanodisks deposited directly on ITO.

© 2020 by the authors. Licensee MDPI, Basel, Switzerland. This article is an open access article distributed under the terms and conditions of the Creative Commons Attribution (CC BY) license (http://creativecommons.org/licenses/by/4.0/).
